# Supplementary material for: An updated vocal repertoire of wild adult bonobos (Pan paniscus)
Source: PLoS One. 2025 Sep 10;20(9):e0330250. doi: 10.1371/journal.pone.0330250 (PMC12422480; doi:10.1371/journal.pone.0330250)
Supplement: S1 File — S1 Table. Sample size of the original call types per individual. S2 Table. Acoustic parameters used in the random forest analysis. The abbreviation of the parameter (which is used in S1 Fig), the full name and a description of the parameter are given. This table is partly taken from (Keen et al. 2021). S3 Analysis. Unsupervised random forest approach. S4 Fig. Influence (mean decrease accuracy) each acoustic parameter has on the random forest model. S5 Fig. Spectrograms of four variants of the “high hoot” call type. These calls were formerly, as “original call types”, categorized as “soft bark”, “bark”, “wieew bark” and “scream bark”. Using our random forest analysis, these former call types are now merged together into a single call category: “high hoots”. S6 Table. Acoustic parameter comparison. The two hitherto performed quantitative analyses of a subset of calls of the vocal repertoire of bonobos ([30] and [29]) and our study used broadly similar, commonly used acoustic parameters for the quantitative acoustic analyses. All three studies used parameters such as duration and parameters related to the distribution of the energy within the call. Whilst we in the study at hand analysed acoustic parameters related to the dominant frequency, Arnaud et al. [30] and Keenan et al. [29] used acoustic features regarding the fundamental frequency. Oftentimes, but not always, the dominant frequency correlates highly with the fundamental frequency. In addition, whilst we used dynamic time warping-related parameters, Arnaud et al. used MFCCs, and Keenan used neither of the two. (ZIP) [file pone.0330250.s001.zip › Supplementary Material/S5_Fig.docx]

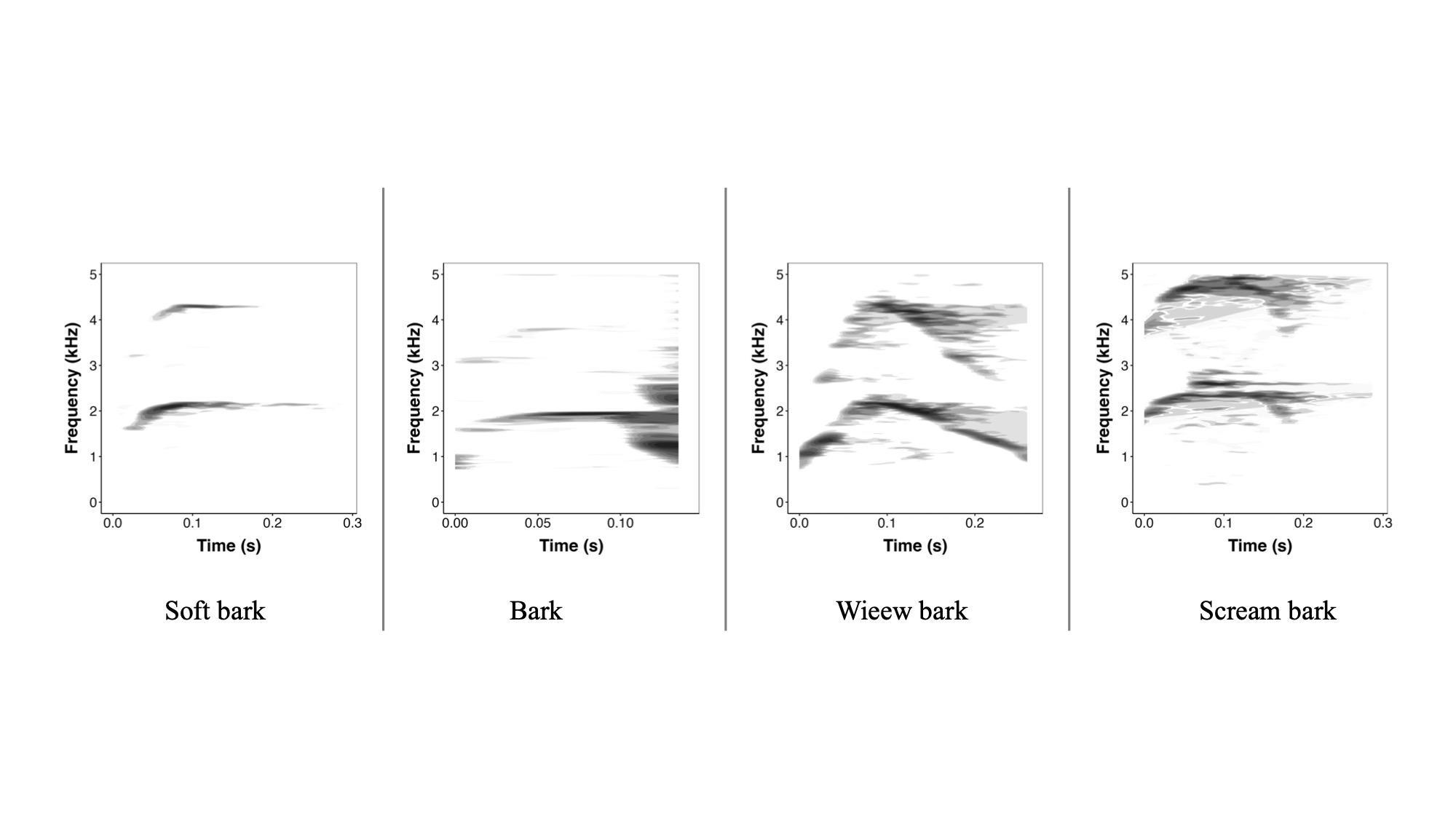


**S5 Fig. Spectrograms of four variants of the “high hoot” call type.**  These calls were formerly, as “original call types”, categorized as “soft bark”, “bark”, “wieew bark” and “scream bark”. Using our random forest analysis, these former call types are now merged together into a single call category: “high hoots”.
